# A reduced VWA domain-containing proteasomal ubiquitin receptor of *Giardia lamblia* localizes to the flagellar pore regions in microtubule-dependent manner

**DOI:** 10.1186/s13071-015-0737-1

**Published:** 2015-02-24

**Authors:** Abhishek Sinha, Shankari Prasad Datta, Atrayee Ray, Srimonti Sarkar

**Affiliations:** Department of Biochemistry, Bose Institute, P 1/12, C. I. T. Road, Scheme – VII M, Kolkata, 700054 West Bengal India

**Keywords:** Proteasome, *Giardia*, Flagella, Ubiquitin, VWA, Rpn10

## Abstract

**Background:**

*Giardia lamblia* switches its lifecycle between trophozoite and cyst forms and the proteasome plays a pivotal role in this switching event. Compared to most model eukaryotes, the proteasome of this parasite has already been documented to have certain variations. This study was undertaken to characterize the ubiquitin receptor, GlRpn10, of the 19S regulatory particle of the *Giardia* proteasome and determine its cellular localization in trophozoites, encysting trophozoites and cysts.

**Method:**

Sequence alignment and domain architecture analyses were performed to characterize GlRpn10. *In vitro* ubiquitin binding assay, functional complementation and biochemical studies verified the protein’s ability to function as ubiquitin receptor in the context of the yeast proteasome. Immunofluorescence localization was performed with antibody against GlRpn10 to determine its distribution in trophozoites, encysting trophozoites and cysts. Real-time PCR and Western blotting were performed to monitor the expression pattern of GlRpn10 during encystation.

**Result:**

GlRpn10 contained a functional ubiquitin interacting motif, which was capable of binding to ubiquitin. Although it contained a truncated VWA domain, it was still capable of partially complementing the function of the yeast Rpn10 orthologue. Apart from localizing to the nucleus and cytosol, GlRpn10 was also present at flagellar pores of trophozoites and this localization was microtubule-dependent. Although there was no change in the cellular levels of GlRpn10 during encystation, its selective distribution at the flagellar pores was absent.

**Conclusion:**

GlRpn10 contains a noncanonical VWA domain that is partially functional in yeast. Besides the expected nuclear and cytosolic distribution, the protein displays microtubule-dependent flagellar pore localization in trophozoites. While the protein remained in the nucleus and cytosol in encysting trophozoites, it could no longer be detected at the flagellar pores. This absence at the flagellar pore regions in encysting trophozoites is likely to involve redistribution of the protein, rather than decreased gene expression or selective protein degradation.

**Electronic supplementary material:**

The online version of this article (doi:10.1186/s13071-015-0737-1) contains supplementary material, which is available to authorized users.

## Background

*Giardia lamblia*, a flagellated parasitic protist, colonizes the gut of its hosts and causes the diarrheal disease giardiasis. The parasite has two distinct morphological stages during its lifecycle: flagellated motile trophozoites and the non-motile cysts. While trophozoites are the disease causing forms, the environmentally-resistant cysts enable the parasite to survive outside the host and the infection cycle commences with ingestion of either water or food contaminated with cysts [1]. Thus, transition from trophozoite to cyst is crucial for disease transmission and this change is brought about by a change in the intracellular proteome of *G. lamblia* [2]. Such changes in intracellular proteome require not only new protein synthesis, but also degradation of existing proteins. Given that the proteasome carries out the bulk of protein degradation in cells [3], investigation of proteasomal function of *Giardia* will be crucial towards understanding stage transition in this protist.

Proteasomes are large macromolecular assemblies that carry out polyubiquitin-dependent protein degradation in a highly-regulated manner, as opposed to the largely unsystematic proteolysis carried out by extracellular proteases. Each proteasome consists of a barrel-shaped 20S core particle (CP) that is composed of proteases and the CP is capped at one or both ends by the 19S regulatory particle (RP). The RP is further subdivided into the base and the lid. The hexameric ring-like base is proximal to the CP and is composed of ATPase subunits, while the lid is distal to the CP and is composed of non-ATPase subunits. The lid is involved in recognition of polyubiquitinated substrates [4]. The presence of the CP of *Giardia* was first reported by Emmerlich *et al.* [5]. Reports also suggested that *Giardia* has the machinery for protein ubiquitination, *viz.* the ubiquitin activating enzyme (E1), ubiquitin conjugating enzymes (E2s), and ubiquitin ligases (E3s) [6]. Recent study by Jerlström-Hultqvist *et al.* [7] has lead to the identification of the RP components of the *Giardia* proteasome by mass spectrometric analyses.

A crucial step in the proteasomal degradation of polyubiquitinated substrates is their recognition by the proteasome. In yeast *Saccharomyces cerevisiae*, the function of polyubiquitinated substrate recognition is primarily carried out by the lid subunits Rpn10 and Rpn13 [8]. The mode of recognition of ubiquitin by these two receptors is different; while Rpn10 binds ubiquitin via the ubiquitin-interacting motif (UIM) [9], Rpn13 recognizes it with the pleckstrin-like receptor of ubiquitin (PRU) domain [10,11]. However, additional ubiquitin recognition factors exist in the yeast proteasome as the double-deletion mutant (*rpn10Δ rpn13Δ*) is viable [8]. Consistently, another proteasome subunit, Rpt5, has been reported to be cross-linked to polyubiquitin chains [12]. Also, shuttle receptors, *viz.* Rad23, Dsk2 and Ddi1, have been identified that have the ability to bind to both ubiquitin and also proteasomal ubiquitin receptors. Thus they serve as adapters for binding of ubiquitinated substrates to the proteasome [13-15]. Given the indispensible requirement for recognition of ubiquitinated substrates by proteasomes, there appears to be multiple factors that have the ability to serve as receptors for ubiquitinated substrates.

A recent study provides an idea regarding the possible subunit composition of *G. lamblia* proteasome wherein the authors performed tandem affinity purification by tagging the putative orthologue of Rpt1, followed by tandem mass spectrophotometry [7]. While this study led to the identification of many of the RP orthologues of the *Giardia* proteasome, it failed to identify Rpn12 and Rpn13. Also the putative Rpn3 of *Giardia* lacked any recognizable PCI domain, which is characteristic of Rpn3 in other eukaryotes [16]. Such deviations in the composition of the proteasome may be consistent with the well-documented evolutionary divergence of *Giardia* [1].

Given the apparent absence of Rpn13, an important ubiquitin receptor in higher eukaryotes, this study has been undertaken to functionally characterize the other major ubiquitin receptor, i.e. the Rpn10 orthologue of *Giardia* (GlRpn10). The results indicate that although GlRpn10 is capable of functioning as an ubiquitin-binding protein, it has variations in the VWA domain that appear to be unique to *Giardia*. Localization studies of this protein in *Giardia* also indicate that apart from the anticipated localization in the cytoplasm and nucleus, the protein is present in the vicinity of the flagellar pores of trophozoites. While this distribution at the flagellar pore is microtubule-dependent and is lost during encystation, the nuclear and cytoplasmic distributions remain unaltered.

## Methods

### Bioinformatic analysis

To search for ubiquitin receptors of the *G. lamblia* proteasome, the sequences of Rpn10 and Rpn13 subunits of *S. cerevisiae*, *B. taurus, H. sapiens* etc. were used as query to BLAST search the *G. lamblia* database (giardiadb.org). The identified sequence was analyzed using Pfam (pfam.sanger.ac.uk) to ascertain the domain composition of the identified protein. GlRpn10 sequence was aligned with other Rpn10/S5a protein sequences of *A. mellifera, H. sapiens, S. cerevisiae*, *M. crystallinum, A. thaliana* and *C. parvum* using CLUSTALW [17] and the multiple sequence alignment was edited and visualized in JALVIEW [18].

### *In vitro* encystations and real-time PCR (RT-PCR)

Trophozoites were grown in TY-I–S 33 medium and encystation was induced according to Kane *et al*. [19]. Cysts were harvested by chilling the tubes on ice and trophozoites that did not undergo encystation were removed by selective lysis, achieved by overnight incubation in distilled water. Purified cysts were lysed by homogenization [19,20]. Total RNA from *G. lamblia* trophozoites, encysting trophozoites and purified cysts was prepared using TRIZOL (Invitrogen) according to manufacturer’s instruction. cDNA was prepared from 2 μg of total RNA using Revertaid Reverse Transcriptase (Thermo Scientific). Real-time PCR was performed using Maxima SYBR green Q-PCR Mastermix (Thermo Scientific) with primers corresponding to the internal sequence of the ORFs (Additional file 1: Table S1). The PCR conditions were as follows: initial denaturation at 95°C for 5 min, followed by 40 cycles of amplification (95°C for 30 s, 60°C for 30 s, 72°C for 30 s).

### Construction of plasmids

For the *in vitro* ubiquitin binding studies, the portion of GL50803_15604 ORF encoding the UIM was PCR amplified using specific primers (Additional file 1: Table S1) and cloned into pET32a (Novagen) using appropriate restriction enzymes (sites italicized in primer sequence given in Additional file 1: Table S1). The tandem-UIM domain of Vps27 was PCR amplified from *S. cerevisiae* genomic DNA using specific primers (Additional file 1: Table S1) and cloned into pET32a. Constructs used for complementation analyses were made using yeast centromeric vector pUS234 containing the *GAL1-10* promoter [21]. ORF GL50803_15604 and *S. cerevisiae RPN10* were PCR amplified using genomic DNA of *G. lamblia* and *S. cerevisiae*, respectively (primers listed in Additional file 1: Table S1). The PCR products were digested with corresponding restriction enzymes and ligated into pUS234 vector. The deletion mutant constructs used in the complementation analyses were created by PCR amplification with the respective primers (Additional file 1: Table S1) and cloning in pUS234. For raising antibody against GlRpn10, GL50803_15604 ORF was PCR amplified using respective primer pair (Additional file 1: Table S1) and cloned in pET32a. All clones were sequenced to confirm the presence of the insert.

### *In vitro* ubiquitin binding assay

The *in vitro* ubiquitin binding experiment described in Shih *et al.* was adopted, but with minor modifications [22]. For this purpose, 6xHis-tagged fusion proteins of *Giardia* and S*accharomyces* UIM domains were overexpressed in *E. coli* BL21 (DE3) cells. After induction, the cells were resuspended in sonication buffer (300 mM NaCl, 50 mM Na_2_HPO_4_, 1 mM PMSF pH 7.0) and lysed by sonication. The UIM fusion proteins, as well as FYVE domain fusion [23], were allowed to bind with the pre-equilibrated Ni-NTA agarose beads (Qiagen) for 1 h at 4°C. The beads were then washed thrice with 20 volumes of 1X PBS containing 50 mM imidazole and 0.1% Triton X-100. For the GST-Ubiquitin (GST-Ub) binding experiment, the fusion proteins immobilized on Ni-NTA beads were incubated with 2 μg of GST-Ub (Boston Biochem) in 1XPBS containing 50 mM imidazole, for 1 h at 4°C. Subsequently the beads were washed thrice with 10 volumes of 1X PBS containing 50 mM imidazole. The bound proteins were then eluted using 1X PBS containing 300 mM imidazole. The amount of eluted protein was measured by Bradford assay [24], loaded equally on a 12% SDS PAGE and subsequently analyzed by Western blotting with anti-GST antibody (Merck Genei).

### Site-directed mutagenesis

Site-directed mutagenesis was carried out using Quickchange Site-directed Mutagenesis kit (Stratagene). Point mutation was inserted into the *Giardia* UIM using PCR based site-directed mutagenesis according to the manufacturer’s instruction using primers listed in Additional file 1: Table S1. The mutation was confirmed by DNA sequencing.

### Complementation analysis in *Saccharomyces cerevisiae*

For complementation analysis in *S. cerevisiae*, *RPN10* was deleted in BY4742 (*MATα his3Δ1 leu2Δ0 lys2Δ0 ura3Δ0*) strain by replacing the sequence with *His3MX6* module, using PCR-based gene deletion [25]. For this purpose, 60 nucleotide-long forward and reverse primers were designed such that 40 nucleotides from each of these primers matched sequences upstream or downstream of the *RPN10* locus and the remaining 20 bases correspond to the *HIS3* gene (Additional file 1: Table S1). The PCR condition was as follows: denaturation at 95°C for 1 min, annealing at 55°C for 1 min and amplification at 72°C for 1.5 min, with 30 cycles of amplification. The resulting PCR product (1376 bp) was gel purified and transformed into BY4742 cells. Transformants were selected by plating on YCM plates containing 2.5 mM 3-amino triazole, but lacking histidine and incubating the plates at 30°C. *rpn10∆* mutants were confirmed by isolating genomic DNA from putative candidates and using the genomic DNA as template in PCR with primers binding to sequence upstream of *RPN10* and within the coding sequence of either *RPN10* or *HIS3* (Additional file 1: Table S1).

The *rpn10∆* and wild-type (BY4742) yeast cells were transformed with constructs carrying GlRpn10, ScRpn10, and various deletion mutants used for the study. For the spot assay, the cells were first grown overnight in liquid YCM medium. Next day, different dilutions of the cells were spotted onto YCM plates lacking uracil, arginine and glucose, but containing 2% galactose, 3% glycerol and 1 μg/ml canavanine. The plates were allowed to grow for 8 days at 30°C to observe the extent of complementation.

### Protein extraction and Western blotting

*G. lamblia* cells were lysed by resuspending in lysis buffer (50 mM Tris-Cl, 100 mM NaCl, 2% SDS, 1% Triton X-100, pH 8.0) and kept on ice for 30 min. Next the protein fraction was collected by centrifugation at 12000 rpm for 10 min. Total protein from BY4742 and *rpn10∆* cells carrying various deletion mutants used for complementation analysis, were prepared by resuspending the cell in suspension buffer (20 ml of buffer contained 1 ml of 1 M Tris-Cl pH 7.5, 200 μl of 0.5 M EDTA, 2 ml of 2.5 M NaCl, 20 μl of NP-40, 30 μl of 1 M DTT, 200 μl of 0.1 M PMSF and 80 μl of protease inhibitor cocktail) and vortexing in presence of glass beads for 10 min at 4°C. Centrifugation was carried-out at 12000 rpm for 15 min and the supernatant was run on gel. For Western blotting, the membrane was blocked with 3% BSA in 1X PBS for 1 h. Antibody against GlRpn10 and anti-ubiquitin antibody (Cell Signaling) was used at 1:800 dilution in 1X PBS carrying 1% BSA. Antibody against 3-PGK (Molecular Probes) was used at 1: 2000 dilution in the same buffer. The membranes were incubated with respective antibody at 4°C overnight. Next the membrane was washed thrice with 1X PBST. Following washing anti-mouse or anti-rabbit peroxidase-conjugated secondary antibody (Santacruz Biotech.) was used at 1:2500 dilution in 1X PBS for 2 h. Membranes were washed as previously described and developed using chemiluminescent substrate (Thermo Scientific).

### Raising polyclonal antibody against GlRpn10 in rabbit

For raising antibody against GlRpn10, the clone containing GlRpn10 in pET32a was first transformed into *E. coli* BL21 (DE3) cells. The fusion protein was overexpressed by induction with 0.1 mM IPTG for 4 h at 37°C. After induction the cells were harvested by centrifugation and resuspended in same sonication buffer that was used for *in vitro* ubiquitin binding experiment. After sonication the cell extract was analyzed by SDS-PAGE to ensure the induction of the desired protein. The purified protein was handed over to BioBharti LifeSciences (Kolkata, India) for raising antibody in rabbit.

### Immunofluorescence studies

Trophozoites, encysting trophozoites and cysts were harvested by chilling the culture tubes on ice for 20 min. The cells were collected by centrifugation at 1000 g for 10 min and washed twice with 1X PBS. The cells were fixed for 20 min with 3% paraformaldehyde in 1X PBS at room temperature. After fixation the cells were collected by centrifugation and treated with 0.1% glycine in 1X PBS for 5 min at room temperature. Subsequently, the cells were collected by centrifugation and permeabilised with 0.1% Triton X-100 in 1X PBS solution (v/v) for 20 min at room temperature. Cysts were permeabilised with 0.2% Triton X-100 in PBS solution for 40 min. After permeabilisation, the cells were blocked with 2% BSA solution in 1X PBS for 1 h. The cells were then incubated overnight with primary antibody at 4°C. Anti-GlRpn10 antibody was used at 1:200 dilution in 1X PBS containing 1% BSA. Cells were harvested by centrifugation and washed thrice in 1X PBS (10 min each wash). Secondary antibody was diluted 1:400 and incubated for 2 h. Before washing away secondary antibody, DAPI was added to the cells at 1 μg/ml concentration and incubated for 15 min. The cells were then washed thrice with 1X PBS. Finally the cell pellet was resuspended in adequate volume of antifade-medium (0.1% p-phenelene-diamine in glycerol) and mounted on glass slides. Confocal laser scanning microscope was used to capture images of cells (Olympus FluoView FV1000).

### Statistical analysis

For the analysis of change in gene expression using real-time PCR, one-way ANOVA was used.

## Results

### Characterization of the UIM of GlRpn10

**Figure 1:**
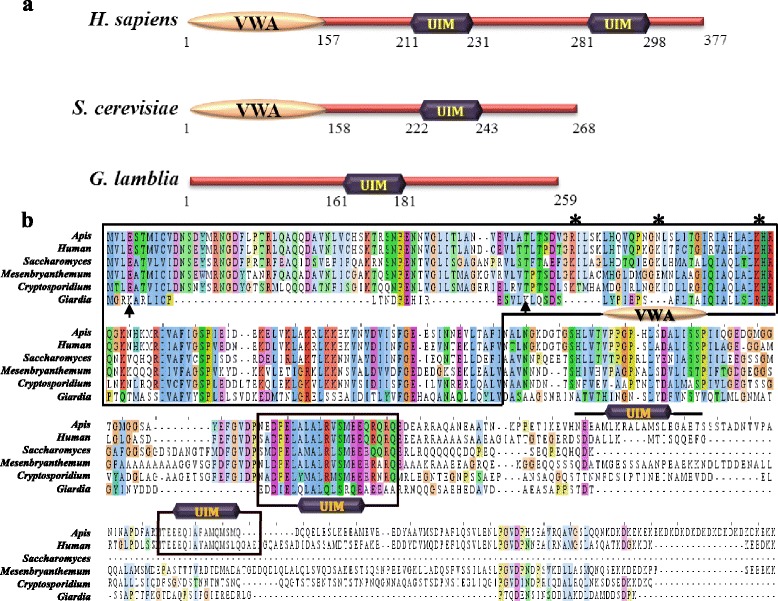
**Domain architecture and sequence alignment of putative GlRpn10** ***.*** **a**. The domain architecture of Rpn10/S5a subunit of *H. sapiens*, *S. cerevisiae* and *G. lamblia.* Numbers indicate the position of domains within the polypeptide chain. **b**. Sequence alignment of GlRpn10 with orthologous sequences from *A. mellifera*, *H. sapiens*, *S. cerevisiae*, *M. crystallinum* and *C. parvum.* Sequences corresponding to the VWA domain and UIM are boxed, except for the third UIM of *A. mellifera*, which is indicated with a bar above the sequence. * represents K residues of the *S. cerevisiae* orthologue that undergoes ubiquitination. Black arrows mark the K residues present close to the N-terminal end of GlRpn10.

**Figure 2:**
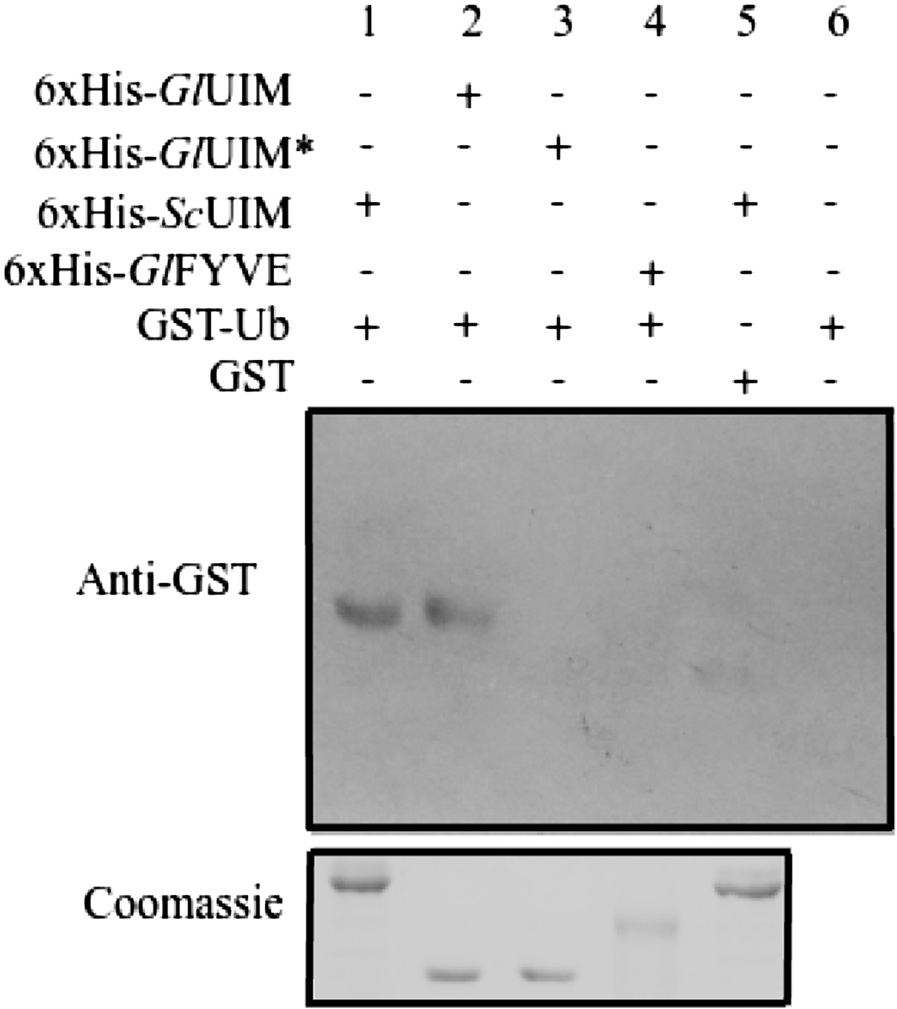
***In vitro*** **ubiquitin binding assay of UIM of GlRpn10 subunit.** UIM of GlRpn10 and ScVps27 were tagged with 6XHis tag. The isolated proteins were immobilized on Ni-NTA agarose beads and allowed to bind GST-tagged ubiquitin. Following elution with imidazole, the eluate was analyzed by Western blotting with anti-GST antibody. *Gl*UIM* represents S → E mutant protein of GlRpn10 UIM. One-fifth of the eluate volume used for the anit-GST blot, was run on a separate gel and stained with coomassie blue.

A recent study has led to the identification of some of the components of the *Giardia* proteasome by performing tandem affinity purification with the tagged Rpt1 orthologue, followed by mass spectrometry [7]. This resulted in the identification of a putative GlRpn10, which is encoded by the ORF GL50803_15604. However, no orthologue of the other ubiquitin receptor, Rpn13, was identified. BLAST searches of the *Giardia* genome with the Rpn13 orthologues from various eukaryotes also failed to identify any putative orthologue of this protein (AS and SS, unpublished results). Even the putative GlRpn10 protein shared very low sequence identity (16.8%) with the *S. cerevisiae* Rpn10 (ScRpn10), thus raising concerns about its capability to function as an ubiquitin receptor of the proteasome. With the aim of functionally characterizing the putative GlRpn10 orthologue, domain architecture analysis of the protein sequence was performed using Pfam and multiple sequence alignment was done to compare the sequence of putative GlRpn10 with sequences of Rpn10 orthologues derived from various eukaryotes like *A. mellifera*, *H. sapiens*, *S. cerevisiae*, *M. crystallinum,* and *C. parvum* (Figure 1a and b). The Rpn10 protein is known to contain two different domains, a VWA domain located towards the N-terminus, and one or more UIMs located after the VWA (Figure 1a). There is variability in the UIM repeat number; while the *S. cerevisiae* orthologue has a single UIM, the human orthologue has two and the fly orthologues (*Drosophila* and *Apis*) have three (Figure 1a and b) [8]. Analysis of the predicted amino acid sequence of GlRpn10 in Pfam indicates that it contains only a single UIM and no other domain (Figure 1a). The predicted UIM of GlRpn10 contains all conserved residues that are characteristic of UIMs. This include the N-terminal acidic amino acids (EDDIE), followed by a large hydrophobic residue (L), an A present two amino acid away, followed by a conserved S at 13^th^ position of the domain (Figure 1b) [26]. This S is crucial for the recognition of ubiquitin and change of this residue to negatively charged amino acid (D or E) hampers the ubiquitin recognition property of UIM [22]. It is known that the UIM domains of S5a (human homolog of Rpn10 that contains two UIMs) have varying affinity for ubiquitin; the UIM of S5a located more towards the N-terminus, has lower affinity for ubiquitin compared to the UIM located after it [27,9]. The UIM of GlRpn10 aligns with the N-terminal UIM of S5a (Figure 1b) rather than the next UIM. Thus, it is possible that the UIM of GlRpn10 may have low affinity for ubiquitin. To test this, the ability of GlRpn10 to bind with ubiquitin was determined by performing *in vitro* ubiquitin-binding assay wherein binding between UIM and GST-ubiquitin (GST-Ub) was ascertained. For this purpose UIM of GlRpn10 was tagged with a 6xHis tag. As positive control, the 6xHis-tagged UIM derived from *S. cerevisiae* Vps27 was used and an unrelated domain of *Giardia*, FYVE, served as negative control [22,23]. While selective retention of GST-Ub was observed for UIM domains derived from Vps27 and GlRpn10, no retention was evident for the 6xHis-tagged FYVE domain (Figure 2, lanes 1, 2 and 4). Lack of binding between Vps27 UIM and GST alone, or between the Ni-NTA resin and GST-Ub served as additional negative controls for this *in vitro* assay (Figure 2, lanes 5 and 6).

To determine if the UIM of GlRpn10 binds to ubiquitin in a manner analogous to other canonical UIMs, the conserved S residue was mutated to E (GlRpn10 UIM*) and its ability to bind GST-Ub was determined. Such a mutation has been previously documented to hamper the ubiquitin binding activity of UIMs [22,26]. It was observed that this mutation completely abolished binding to GST-Ub (Figure 2, lane 3). Taken together, the above results demonstrate that although GlRpn10 contains only a single UIM, this motif has the ability to bind to ubiquitin in a manner similar to other well-characterized UIMs.

### GlRpn10 contains a truncated VWA domain

In contrast to the canonical UIM domain, Pfam analysis of the GlRpn10 failed to predict the presence of a VWA domain (Figure 1a). This is unusual given that the VWA domain is important for the functioning of Rpn10. Studies with ScRpn10 indicate that the VWA domain of this protein regulates its activity. Internal K residues present in the VWA domain (marked with * in Figure 1b) undergo monoubiquitination, a modification that regulates the ability of the ScRpn10 UIM to recognize and recruit ubiquitinated substrates to the proteasome [28]. Interestingly, perusal of the sequence alignment indicated that while GlRpn10 shared considerable stretches of sequence similarity with the C-terminal end of the VWA domains present in the Rpn10 orthologues included in this study, it lacked sequences at the N-terminal end, which are present in all the other orthologues (Figure 1b). This deletion at the N-terminal end is probably why Pfam analysis did not result in the identification of a VWA domain in GlRpn10. Absence of a full-length VWA domain raises questions regarding the ability of the identified GlRpn10 to function in the context of the proteasome. To address this issue, functional complementation analysis was performed in *S. cerevisiae* to determine if GlRpn10 can substitute for the yeast Rpn10 protein.

**Figure 3:**
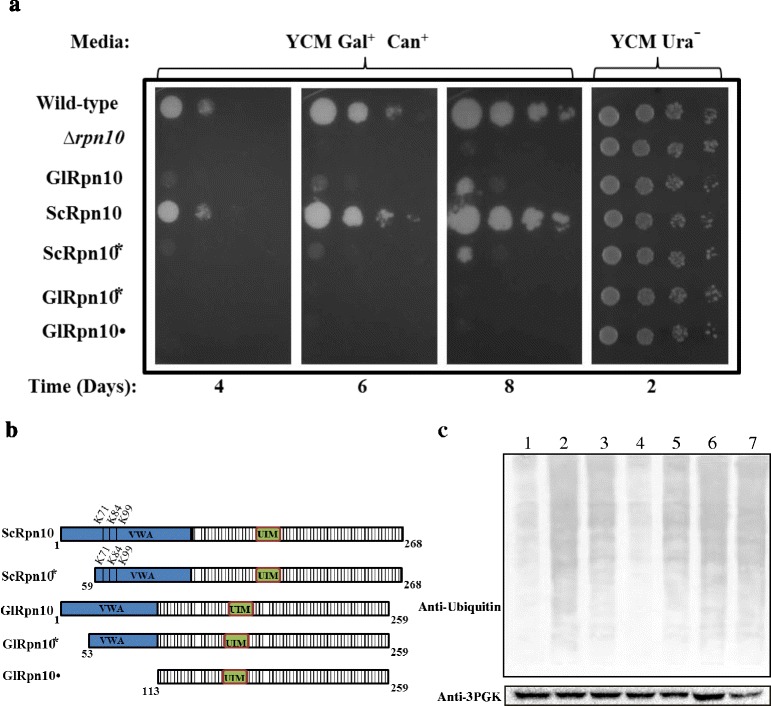
**Functional complementation with GlRpn10. (a)** *S. cerevisiae rpn10∆* strain was transformed individually with each of the constructs expressing the proteins shown in Panel b. The growth of these transformed yeast cells was monitored by spot test using serial dilutions on YCM plates lacking uracil and containing galactose and canavanine. To ensure that equal number of cells have been used, spotting was also done on YCM plates lacking uracil and containing glucose. All the plates were incubated at 30 °C. **(b)** Schematic diagrams of GlRpn10, ScRpn10, and different deletion variants of these two proteins. The regions corresponding to the two domains, VWA and the UIM, are denoted in blue and green respectively. The K residues within the VWA domain of ScRpn10 are marked and their respective positions are indicated above. **(c)** Western blot using anti-ubiquitin antibody of the total cell extract of wild-type, *rpn10∆* and *rpn10∆* transformed with the above mentioned constructs. The composition of the growth medium is same as given in **(a)** above, except that these transformants were grown in liquid medium. Extracts were loaded in the following order: lane 1, Wild-type transformed with vector; lane 2, *rpn10∆* transformed with vector; lane 3, *rpn10∆* cells expressing GlRpn10; lane 4, *rpn10∆* cells expressing ScRpn10; lane 5, *rpn10∆* cells expressing ScRpn10*; lane 6, *rpn10∆* cells expressing GlRpn10* and lane 7, *rpn10∆* cells expressing GlRpn10•. 3-PGK was used as loading control.

*RPN10* is a non-essential gene as the growth of yeast mutants with deletion of chromosomal *RPN10* (*rpn10Δ*) is indistinguishable from that of wild-type cells at 30°C. However, when the cells are subjected to stress by growing them in the presence of amino acid analogues, such as canavanine (analogue of arginine), *rpn10∆* cells fail to grow at 30°C [29]. This is because replacement of arginine with canavanine in the growth media results in production of defective proteins, which leads to increase in the misfolded protein load within the cell. Since this situation can only be countered with a fully functional proteasome, ScRpn10 becomes essential for survival in the presence of canavanine. For the functional complementation study, *RPN10* was deleted from yeast genome and as expected, the mutant was unable to grow on YCM plates containing canavanine (Figure 3a). Growth of this mutant was restored to wild-type levels when ScRpn10 was expressed under the control of a galactose-inducible promoter (*GAL1-10* promoter). Expression of GlRpn10 resulted in partial rescue of the growth phenotype of *rpn10∆* cells (Figure 3a). This partial growth rescue phenotype of GlRpn10 may result from the absence of sequences from the N-terminal end of the GlRpn10 protein (Figure 1b) as a previous study has shown that a deletion of 61 amino acids from the N-terminus of ScRpn10 results in growth defects in the presence of amino acid analogues canavanine and *p*-flurophenylalanine [30]. The sequence alignment indicates that region of similarity between ScRpn10 and GlRpn10 starts around the 60^th^ residue of the yeast protein (VLSTF sequence in ScRpn10) (Figure 1b). Using the present assay conditions, a deletion of the first 58 residues of ScRpn10 (ScRpn10*) also resulted in partial rescue of the growth phenotype of *rpn10∆* and the extent of the partial rescue was similar to that observed with GlRpn10 (Figure 3a, compare GlRpn10 and ScRpn10*). Thus it may be concluded that the identified GlRpn10 protein is most likely to function as a component of the yeast proteasome. However, it is not fully functional as it lacks the N-terminal segment of the VWA domain.

As previously mentioned, internal K residues of yeast VWA domain are subjected to ubiquitination and this ubiquitin modification plays regulatory role by modulating the ubiquitinated substrate recognition capability of Rpn10 [28]. Although GlRpn10 lacks K residues at the corresponding positions, two K residues are present towards its N-terminus (marked with arrows in Figure 1b). To understand if the truncated VWA domain plays a role in the functional complementation process, it was next determined if the region of GlRpn10 containing these K residues has any role in the partial complementation phenotype. The portion of the VWA domain containing these K residues was truncated (GlRpn10* in Figure 3b). Expression of GlRpn10* completely failed to rescue the growth defect phenotype of *rpn10Δ* mutants, as did a GlRpn10 variant that completely lacked the VWA domain (GlRpn10•, Figure 3a and b). Therefore, although a substantial portion of the VWA domain is missing from GlRpn10, the domain is still essential and thus may retain the ability to discharge some of the functions of the full-length version of the domain.

A biochemical approach was used to validate the results of the complementation studies. In absence of functional Rpn10, yeast cells accumulate ubiquitinated proteins [29]. The overall levels of ubiquitinated proteins present in the cells harboring all the above-mentioned Rpn10 variations were determined. Western blotting with anti-ubiquitin antibody showed that the levels of ubiquitinated proteins, relative to wild-type cells, increased when *rpn10Δ* mutants were grown in the presence of canavanine (Figure 3c, lanes 1 and 2). While the amount of ubiquitinated proteins was restored to wild-type levels with the expression of ScRpn10, expression of GlRpn10 resulted in only partial reduction (Figure 3c, lanes 3 and 4). Expression of ScRpn10* also reduced the ubiquitinated proteins to levels comparable to that of GlRpn10 (Figure 3c, lane 5). However, the expression of GlRpn10* and GlRpn10• failed to cause any detectable reduction of the cellular ubiquitinated protein levels compared to that observed in *rpn10Δ* mutants (Figure 3c, lanes 6 and 7). Thus the cellular ubiquitin levels are consistent with the growth of these mutants on canavanine plates. Therefore, both genetic and biochemical approaches indicate the GlRpn10 is capable of functioning in context of proteasome and it encodes a reduced VWA domain that is only partially functional compared to the yeast VWA domain.

### Unique distribution of GlRpn10 in trophozoites

**Figure 4:**
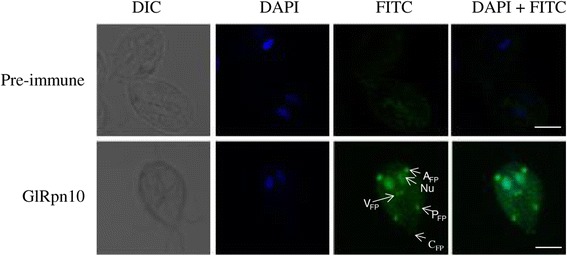
**Immunofluorescence microscopy of** ***Giardia*** **trophozoites with anti-GlRpn10 antibody.** Immunofluorescence was performed with antibody against GlRpn10 raised in rabbit. FITC-conjugated anti-rabbit antibody was used as secondary antibody and cells were observed under confocal laser scanning microscope. DAPI was used to label DNA. The top panel shows cells where pre-immune antiserum was used instead of the primary antibody. Arrows point to the following regions: A_FP_: anterior flagellar pore, P_FP_: posteriorlateral flagellar pore, V_FP_: ventral flagellar pore, C_FP_: caudal flagellar pore and Nu: nucleus. Bar represents 5 μm.

Stefanic *et al*. have previously reported the CP subunit component, Glα7, has both nuclear and cytoplasmic distribution [31]. To determine if GlRpn10 has a similar cellular distribution, polyclonal antibody was raised against the recombinant GlRpn10 in rabbit. The antibody recognized a protein of approximately 28 kDa that is not detectable with the pre-immune sera (Additional file 2: Figure S1). This size is consistent with the predicted size of GlRpn10, which is composed of 259 amino acids. This antibody was used for performing immunofluorescence experiment and the cells were observed using confocal laser scanning microscopy. Consistent with the previous report for Glα7, both nuclear and cytoplasmic pools of GlRpn10 was observed (Figure 4, bottom panel and Additional file 3: Video 1). Additionally, GlRpn10 also localized to eight bright spots that are located at or near the cell periphery (Figure 4, bottom panel). These spots appeared at regions of the cell periphery from where the anterior, posteriorlateral, ventral and caudal flagella emerge, i.e. the flagellar pores. The intensity of the signal was the maximum at the anterior flagellar pores and least at the caudal flagellar pores. Thus, in addition to the expected nuclear and cytoplasmic distribution, GlRpn10 also has a unique localization at the flagellar pore regions. Given that the components of CP and the base of the RP do not localize at flagellar pores [31], this distribution may arise from a pool of GlRpn10 that is not associated with the proteasome.

### Localization of GlRpn10 to the flagellar pores is microtubule-dependent

**Figure 5:**
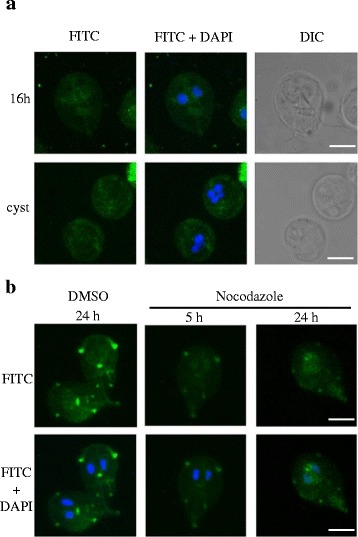
**Distribution of GlRpn10 during different stages of the** ***G. lamblia*** **lifecycle and upon nocodazole treatment. (a)** Localization of GlRpn10 in encysting *G. lamblia* (16 h after induction of encystation) and in cyst was determined by immunofluorescence as mentioned in Figure 4. **(b)** Distribution of GlRpn10 in trophozoites 5 h and 24 h after nocodazole treatment. In both cases DAPI was used to stain DNA. Bar represents 5 μm.

As GlRpn10 localized to the flagellar pores, the role of the flagella, if any, in such a selective localization was investigated. Towards this goal, the distribution of GlRpn10 was determined in encysting trophozoites and cysts as flagella start to regress during encystation and are completely internalized in cysts [1]. Trophozoites were induced to undergo encystation with bovine bile and the localization of GlRpn10 was determined in encysting trophozoites (16 h post-induction) and cysts. In the encysting trophozoites, it was observed that while the signal for GlRpn10 persisted in the cytoplasm and the nucleus, its distribution at the flagellar pore region was not evident (Figure 5a). In the tetranucleated cysts, GlRpn10 was distributed in the cytoplasm (Figure 5a). Thus, there appears to be a selective reduction of the GlRpn10 signal only at the flagellar pores of encysting cells.

Since GlRpn10 localizes at the flagellar pores and flagellar pores are enriched in microtubular structures [32], it was next determined if this localization is dependent on microtubules. As nocodazole hampers microtubule polymerization, it was determined if the distribution of GlRpn10 is altered upon treatment with this drug. Based on previous studies, trophozoites were exposed to 10 μM nocodazole for 5 h and 24 h; DMSO treatment served as control [32,33]. Following this treatment, GlRpn10 was immunolocalized and it was observed that, in comparison to DMSO-treated control cells, the presence of GlRpn10 at the flagellar pores decreased in nocodazole-treated cells (Figure 5b). While the distribution in the nucleus and cytoplasm remained unaltered, a time-dependent decrease in intensity was observed at the flagellar pore regions in the nocodazole-treated cells. Staining of alpha tubulin revealed the depolymerization of microtubule structures, such as the median body, in nocodazole-treated cells (data not shown). Taken together, these results indicate that the selective distribution of this protein at the flagellar pore region is microtubule dependent.

### Expression pattern of *glrpn10* during encystation in cysts

**Figure 6:**
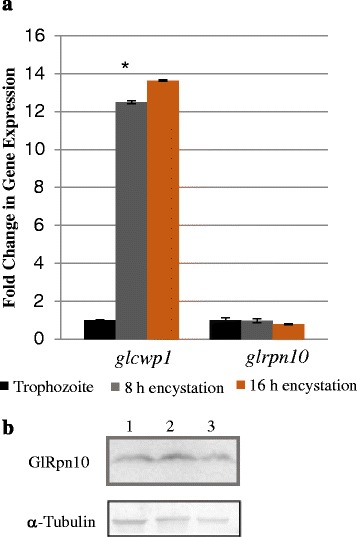
**Expression pattern of** ***glrpn10*** **mRNA and GlRpn10 during encystation. (a)** Expression of genes encoding *gl*c*wp1* and *glrpn10* in trophozoites and encysting trophozoites (8 h and 16 h) was determined by real-time PCR. Experiments were performed in triplicate. The real-time analysis was validated using one-way ANOVA analysis. * represents P value of < 0.001. **(b)** Western blot with anti-GlRpn10 antibody in trophozoites (lane 1) and encysting trophozoites (8 h, lane 2 and 16 h, lane 3). α-tubulin was used as a loading control.

The disappearance of GlRpn10 from the base of the flagella of encysting cells may also result from decreased expression of this protein. To determine if the observed selective distribution of GlRpn10 at the flagellar pores of trophozoites and the subsequent selective disappearance from this location during the process of encystation entails any change in the cellular levels of GlRpn10, the expression pattern of the encoding gene was monitored by real-time PCR and modulation of the protein levels was determined by Western blotting. For the real-time PCR analysis, cDNA was prepared from trophozoites and encysting trophozoites (8 h and 16 h after induction of encystation). Expression profile of *cwp1* served as a positive control [34]. The results show that while the expression of *cwp1* was upregulated several fold during encystation, there was no significant change of *glrpn10* expression in either 8 h or 16 h encysting cells, relative to that of trophozoites (Figure 6a). Western blotting of protein extracts corresponding to these time points also indicated that there was no detectable change in the levels of the protein (Figure 6b). This observation is consistent with the lack of change in the expression of the CP subunits during encystation [35]. Thus, based on analyses of the gene expression and protein levels in trophozoites and encysting trophozoites, it may be concluded that the decreased localization of GlRpn10 at the flagellar pore regions during encystation is likely to involve redistribution of the protein from these locations, rather than decreased gene expression or protein degradation.

## Discussion

Regulated intracellular protein degradation is vital for cell survival and thus, the proteasomal system exists in all the three domains of life. The proteasomes of parasites may also be important in host-parasite interactions as it has been documented that the gene encoding the proteasome subunit beta type-7 is upregulated in the intestinal infected larvae of the nematode parasite *Trichinella spiralis*, compared to the levels present in its muscle larvae form [36]. This study has focused on GlRpn10, a receptor for ubiquitin, which is part of the RP of *Giardia* proteasome. Structure-function studies indicate that while the CPs from bacteria, archaea and eukaryotes are fairly similar, the RP of actinobacteria and archaea are much less complex compared to their eukaryotic counterpart [37]. Interestingly, the results of this study and another recent report [7] indicates that, compared to those present in most model eukaryotes, the *Giardia* proteasome may also be composed of fewer RP subunits as both studies failed to identify proteins orthologous to Rpn12 and Rpn13 in the *G. lamblia* genome. Thus this is yet another instance of simplification of cellular machinery in *G. lamblia*. Other examples of such simplified machinery of this parasite include fewer components participating in Pol II-dependent transcription and also cap-dependent translation [1].

Results of the current study also indicate that lower complexity of the *Giardia* proteasome may be extended to even individual subunits as GlRpn10 contains a reduced VWA domain. The VWA domain is present in bacteria, archaea and eukaryotes and proteins containing it usually function in multi-protein complexes [38]. Although such proteins may be either intracellular or extracellular, the intracellular orthologues are considered to be more ancient in evolutionary terms and these are involved in processes such as transcription, DNA repair, ribosomal and membrane transport and also the proteasome [38]. Structural studies show that the VWA domain is composed of a central β sheet that is sandwiched in between two sets of α helices [39]. The β sheet is composed of six strands, of which only one strand, located at the edge, is anti-parallel. All VWA domains identified thus far are predicted to have this structure. Such full-length VWA domains are even present in three other *Giardia* proteins, *viz.* the orthologues of transcription factor TFIIHp44 (Gl50803_15000), Sec23 (Gl50803_9376) and Sec24 (Gl50803_17065) (unpublished results). Thus, the observed reduction of the VWA domain only in the case of the GlRpn10 raises the possibility of altered assembly of the *Giardia* RP. However, it is known that a truncated ScRpn10 missing a similar segment of the protein (first 60 amino acids) is not only capable of binding to ubiquitin but is also incorporated into the proteasome [30]. This lends support to the conclusion that even though the GlRpn10 protein harbors a reduced VWA domain, it is capable of incorporating into the proteasome. Further studies are necessary to determine if the absence of sequences at the N-terminal end of GlRpn10 results in a difference in the overall stability of the proteasome. Thus, the present study has identified a new natural variant of the VWA domain whose biological properties are similar to that of the truncations generated from full-length VWA domains [30]. Preliminary homology modeling studies of the smaller VWA domain of GlRpn10 indicated that this truncation leads to the absence of a α-helix and two β-strands (Additional file 4: Figure S2). Also, the anti-parallel edge β − strand may or may not be present. Interestingly, in this model, the two N-terminal K residues of GlRpn10 are present not only on the same face of the domain but also on the same secondary structure elements as the K residues of ScRpn10 that undergo monoubiquitination. As previously mentioned, this monoubiquitination serves to regulate the ability of Rpn10 to bind to ubiquitinated substrates. Thus, these K residues of the GlRpn10 may also undergo ubiquitination and future studies are likely to shed light on the functional importance of these K residues.

Results of this study also indicate that the localization of GlRpn10 to the flagellar pore region is dependent on microtubules (Figure 5). The functional significance of this localization remains to be determined. While the role of ubiquitination in degradation of tubulin is well documented (for example, degradation of tubulin by the E3 ligase parkin), the connection between Rpn10/S5a and tubulin is indirect [40]. Genetic studies show that Rpn10 is a negative regulator of ID1, a transcription inhibitor whose ectopic expression results in increased number of centrosomes; this increased-centrosome phenotype is suppressed by the ectopic expression of S5a [41]. Also, the centrosomal marker, γ-tubulin, has been shown to colocalize with 20S, 19S, ubiquitin and parkin in HEK293 cells [42] and S5a cofractionated with γ-tubulin [43]. In fact S5a has been shown to play a role in linking proteasomes to the centrosomes [43]. But, given the fact that γ-tubulin localizes to the minus ends of microtubules, the localization of GlRpn10 to the flagellar pore region is unlikely to be mediated by its interaction with γ-tubulin as minus ends of microtubules are unlikely to be present at this location. However, it may be noted that γ-tubulin has been found to localize to discrete dots in the subpellicular microtubular array of *Leishmania* [44]. Thus it is not possible to completely rule out the fact that the flagellar pore regions of *Giardia* represent equivalent regions of the subpellicular microtubules. Taken together, it is possible that GlRpn10 may associate with microtubules, either directly or indirectly. Towards this detergent co-fractionation experiment was performed but it failed to establish significant association between GlRpn10 and α-tubulin (Additional file 5: Figure S3). However, it may be worth noting that only a small fraction of GlRpn10 is present at the flagellar pores, with the bulk of the protein being distributed to the cytoplasm and the nucleus. Thus, even if there is association of this minor pool of GlRpn10 with the tubulin cytoskeleton, a biochemical assay such as detergent co-fractionation, may not be sensitive enough to detect this. It is also possible that GlRpn10 is part of a complex that associates with the microtubules and there is no direct interaction between the two.

Another open question is if the pool of GlRpn10 localizing to the flagellar pore regions is extra-proteasomal. The possibility of extra-proteasomal GlRpn10 is supported by the observation that the CP component Glα7 does not display flagellar pore localization [31]. Also pools of extra-proteasomal Rpn10 have been identified in *S. cerevisiae* [45]. Intriguingly, a protein involved in translation, eIF4E2, as well as microtubule associated protein EB1 also exhibit similar localization at the flagellar pore region in *Giardia* [46,47]. Thus, the flagellar pore regions may be the location of regulatory events that are yet to be characterized. Therefore, understanding the functional significance of protein localization to this novel subcellular domain of *Giardia* is likely to be an important area of future study.

## Conclusions

This study presents the functional characterization of the ubiquitin receptor of the proteasome of *G. lamblia*, using *in vitro* and *in vivo* approaches. GlRpn10 contains only a single UIM domain, which has the ability to bind to ubiquitin *in vitro*. However, a substantial portion of the VWA domain is missing from GlRpn10. Even with the truncated VWA domain, the protein can still function in the context of the proteasome, indicating that it retains the ability to discharge some of the functions of the full-length version of the domain. This study is the first to identify a new natural variant of the VWA domain. The localization of this protein also indicates that besides the expected nuclear and cytoplasmic distribution, it is also present at the flagellar pore regions and this localization is microtubule-dependent. The flagellar pore localization could not be detected in encysting trophozoites.

## Additional files

Additional file 1: Table S1. Sequences of primers used in this study. Additional file 2: Figure S1. Western blot performed with the antibody raised against GlRpn10. Whole cell extracts of *G. lamblia* trophozoite was subjected to SDS-PAGE and probed with either pre-immune serum (lane 1) or the antibody raised against GlRpn10 (lane 2). Use of the antibody resulted in the detection of a band corresponding to the predicted size of GlRpn10 (28 kDa). This band was not observed when pre-immune serum was used. Additional file 3: Video 1. 3D-reconstruction of GlRpn10 localization in trophozoites. Additional file 4: Figure S2. Homology-modeled structures of VWA domains of ScRpn10 and GlRpn10. The sequences of GlRpn10 and ScRpn10 were used as input in Phyre2 for homology modeling. The modeling is based on the single highest scoring template chosen from all known structures. **(a)** Predicted structure of the VWA domain of GlRpn10, based on the structure of the *S. pombe* Rpn10 VWA domain (PDB code: 2X5N). Blue arrows point to the K residues within the reduced VWA domain of GlRpn10, the side chains of which are also shown in blue. Yellow coloured loop denotes the possible region where a beta-sheet may be formed as predicted by the secondary structure prediction tool of Phyre2. **(b)** Predicted structure of the VWA domain of ScRpn10, based on its own structure (PDB code: 4CR2). The K residues within ScRpn10 VWA domain that undergo monoubiquitination are marked as stated above. **(c)** Superimposition of the structures given in **(a)** & **(b)**. Additional file 5: Figure S3. Fractionation with buffer containing 0.5% Triton X-100 to determine association of Rpn10 with cytoskeleton. The Triton X-100-soluble fraction containing the soluble cytoplasmic, plasma membrane and organellar (membrane and lumen) proteins (lane 1) were separated from the Triton X-100-insoluble fraction containing cytoskeletal proteins (lane 3). The detergent-resistant pellet was resuspended by douncing with a Teflon homogenizer, followed by centrifugation. The resulting pellet is the Triton X-100-insoluble fraction. The supernatant, obtained after homogenization and subsequent centrifugation, was also loaded onto the gel (lane 2) to ensure the purity of the two extracts. Absence of any GlRpn10 in the Triton X-100-insoluble fraction indicates that this protein does not directly associate with the microtubules.

## Abbreviations

BLAST: Basic local alignment search tool
CP: Core particle
RP: Regulatory particle
UIM: Ubiquitin interacting motif
VWA: von Willebrand factor type A
GST-Ub: Glutathione S-transferase-Ubiquitin
DMSO: di-methyl sulfoxide
