# Supplementary material for: A reduced VWA domain-containing proteasomal ubiquitin receptor of Giardia lamblia localizes to the flagellar pore regions in microtubule-dependent manner
Source: Parasit Vectors. 2015 Feb 24;8:120. doi: 10.1186/s13071-015-0737-1 (PMC4352536; doi:10.1186/s13071-015-0737-1)
Supplement: Additional file 1: Table S1. — Sequences of primers used in this study. [file 13071_2015_737_MOESM1_ESM.doc]

Supplementary Table 1: Sequences of primers used in this study

| **Primer** | **Sequence 5’→3’** | **Purpose** |
| --- | --- | --- |
| AS1Fwd | CG*GGATCC*ATCAATTACGACGACGATG | Forward primer for cloning of *Giardia* UIM in pJET1/2 and pET32a(+) and real-time PCR |
| AS1Rvse | *AAGCTT*TGCACTACCTTGCTGGTTGCG | Reverse primer for cloning of *Giardia* UIM in pJET1/2 and pET32a(+) and real-time PCR |
| AS2Fwd | AGA*GGATCC*GACAGGGACTACTCAAC | Forward primer for cloning of Vps27 tandem UIM in pET32a(+) |
| AS2Rvse | CTG*GAATTC*TTGCATCTGTCTAGAGGC | Reverse primer for cloning of Vps27 tandem UIM in pET32a(+) |
| AS3Fwd | CG*AAGCTT*TTGCATCTGTTGTTGG | Forward primer for cloning of GlRpn10 in pUS234 |
| AS3Rvse | G*AAGCTT*GGCAGAAACAGTATGGG | Reverse primer for cloning of GlRpn10, GlRpn10⃰ , GlRpn10• in pUS234 and pET32a |
| AS4Fwd | GACA*AAGCTT*GCAGTAACCGCACAATG | Forward primer for cloning of ScRpn10 in pUS234 |
| AS4Rvse | GGT*AAGCTT*TAGTCTTGGTGTTGTTCAGG | Reverse primer for cloning of ScRpn10 in pUS234 |
| AS5Fwd | *AAGCTT*ACAATGGTAGGTAAGCTGCATATGG | Forward primer for cloning of ScRpn10⃰ in pGEMT and later pUS234 |
| AS5Rvse | AGCC*AAGCTT*CTATTTGTCTTGGTGTTG | Reverse primer for cloning of ScRpn10⃰ in pGEMT and later pUS234 |
| AS6 Fwd | *GGATCC*GGAATGGGGCCAACCCAAACGATGG | Forward primer for cloning of GlRpn10⃰ in pGEMT and later pUS234 |
| AS7Fwd | *GGATCC*GGAATGGGGGTGGATGCTTCGGCT | Forward primer for cloning of GlRpn10• in pGEMT and later pUS234 |
| AS8Fwd | GAAGGGAAAAGTCAATAAATAACTCAGCAGTAACCGCACACGGATCCCCGGGTTAATTAA | Forward primer for deletion of *RPN10* |
| AS8Fwd | TGTAACACTACTATTTGTCTTGGTGTTGTTCAGGCTGTTCAGAATTCGAGCTCGTTTAAAC | Reverse primer for deletion of *RPN10* |
| AS9Fwd | CAGTTGGCTTTGCAGCTGGATAGACAAGAGGCGGAGGAAGC | Forward primer for site-directed mutagenesis of GlRpn10 UIM domain |
| AS9Rvse | CGCCTCTTGTCTATCCAGCTGCAAAGCCAACTGGAGCTCAA | Reverse primer for site-directed mutagenesis of GlRpn10 UIM domain |
| AS10 Fwd | AAGCGGATCCCTTGCATCTGT | Forward primer for cloning GlRpn10 in pET32a |
| Rpn10 selection F | GCTCATATTGCGTGGAGAAGGGCC | Forward primer for deletion confirmation in *∆rpn10* candidates |
| Rpn10 selection R | TGGCCATATGCAGCTTACCCTCG | Reverse primer for deletion confirmation in *∆rpn10* candidates from within the *RPN10* gene |
| pFA selection R | ATGTTCGGATGTGATGTG | Reverse primer for deletion confirmation in *∆rpn10* candidates from within the *HIS3* gene |
