# Supplementary material for: A reduced VWA domain-containing proteasomal ubiquitin receptor of Giardia lamblia localizes to the flagellar pore regions in microtubule-dependent manner
Source: Parasit Vectors. 2015 Feb 24;8:120. doi: 10.1186/s13071-015-0737-1 (PMC4352536; doi:10.1186/s13071-015-0737-1)
Supplement: Additional file 2: Figure S1. — Western blot performed with the antibody raised against GlRpn10. Whole cell extracts of G. lamblia trophozoite was subjected to SDS-PAGE and probed with either pre-immune serum (lane 1) or the antibody raised against GlRpn10 (lane 2). Use of the antibody resulted in the detection of a band corresponding to the predicted size of GlRpn10 (28 kDa). This band was not observed when pre-immune serum was used. [file 13071_2015_737_MOESM2_ESM.pptx]

## Slide 1
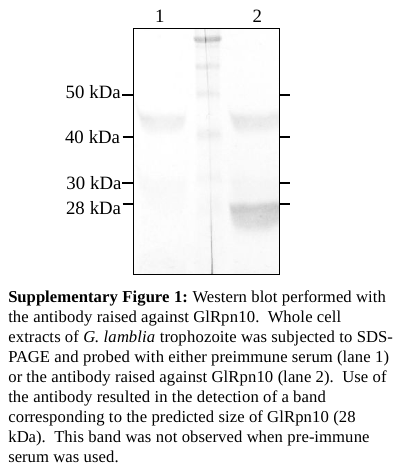

1
2
50 kDa
40 kDa
30 kDa
28 kDa
Supplementary Figure 1: Western blot performed with the antibody raised against GlRpn10. Whole cell extracts of G. lamblia trophozoite was subjected to SDS-PAGE and probed with either preimmune serum (lane 1) or the antibody raised against GlRpn10 (lane 2). Use of the antibody resulted in the detection of a band corresponding to the predicted size of GlRpn10 (28 kDa). This band was not observed when pre-immune serum was used.
