# Supplementary material for: A reduced VWA domain-containing proteasomal ubiquitin receptor of Giardia lamblia localizes to the flagellar pore regions in microtubule-dependent manner
Source: Parasit Vectors. 2015 Feb 24;8:120. doi: 10.1186/s13071-015-0737-1 (PMC4352536; doi:10.1186/s13071-015-0737-1)
Supplement: Additional file 4: Figure S2. — Homology-modeled structures of VWA domains of ScRpn10 and GlRpn10. The sequences of GlRpn10 and ScRpn10 were used as input in Phyre2 for homology modeling. The modeling is based on the single highest scoring template chosen from all known structures. (a) Predicted structure of the VWA domain of GlRpn10, based on the structure of the S. pombe Rpn10 VWA domain (PDB code: 2X5N). Blue arrows point to the K residues within the reduced VWA domain of GlRpn10, the side chains of which are also shown in blue. Yellow coloured loop denotes the possible region where a beta-sheet may be formed as predicted by the secondary structure prediction tool of Phyre2. (b) Predicted structure of the VWA domain of ScRpn10, based on its own structure (PDB code: 4CR2). The K residues within ScRpn10 VWA domain that undergo monoubiquitination are marked as stated above. (c) Superimposition of the structures given in (a) & (b). [file 13071_2015_737_MOESM4_ESM.ppt]

## Slide 1
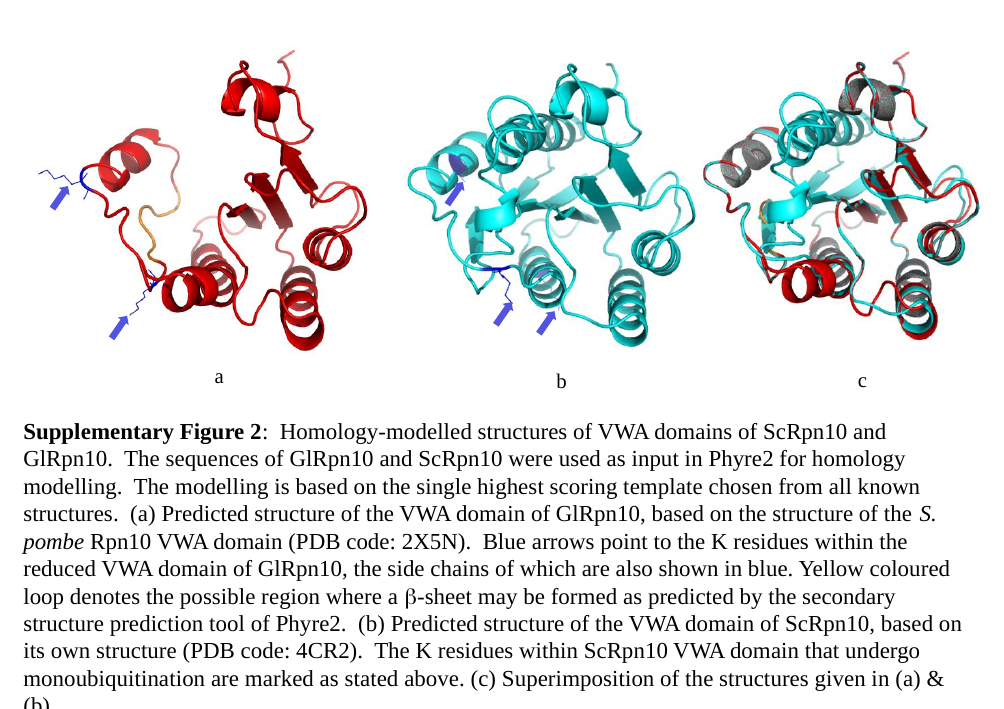

a
c
b
Supplementary Figure 2: Homology-modelled structures of VWA domains of ScRpn10 and GlRpn10. The sequences of GlRpn10 and ScRpn10 were used as input in Phyre2 for homology modelling. The modelling is based on the single highest scoring template chosen from all known structures. (a) Predicted structure of the VWA domain of GlRpn10, based on the structure of the S. pombe Rpn10 VWA domain (PDB code: 2X5N). Blue arrows point to the K residues within the reduced VWA domain of GlRpn10, the side chains of which are also shown in blue. Yellow coloured loop denotes the possible region where a β-sheet may be formed as predicted by the secondary structure prediction tool of Phyre2. (b) Predicted structure of the VWA domain of ScRpn10, based on its own structure (PDB code: 4CR2). The K residues within ScRpn10 VWA domain that undergo monoubiquitination are marked as stated above. (c) Superimposition of the structures given in (a) & (b).
