# Supplementary material for: A reduced VWA domain-containing proteasomal ubiquitin receptor of Giardia lamblia localizes to the flagellar pore regions in microtubule-dependent manner
Source: Parasit Vectors. 2015 Feb 24;8:120. doi: 10.1186/s13071-015-0737-1 (PMC4352536; doi:10.1186/s13071-015-0737-1)
Supplement: Additional file 5: Figure S3. — Fractionation with buffer containing 0.5% Triton X-100 to determine association of Rpn10 with cytoskeleton. The Triton X-100-soluble fraction containing the soluble cytoplasmic, plasma membrane and organellar (membrane and lumen) proteins (lane 1) were separated from the Triton X-100-insoluble fraction containing cytoskeletal proteins (lane 3). The detergent-resistant pellet was resuspended by douncing with a Teflon homogenizer, followed by centrifugation. The resulting pellet is the Triton X-100-insoluble fraction. The supernatant, obtained after homogenization and subsequent centrifugation, was also loaded onto the gel (lane 2) to ensure the purity of the two extracts. Absence of any GlRpn10 in the Triton X-100-insoluble fraction indicates that this protein does not directly associate with the microtubules. [file 13071_2015_737_MOESM5_ESM.pptx]

## Slide 1
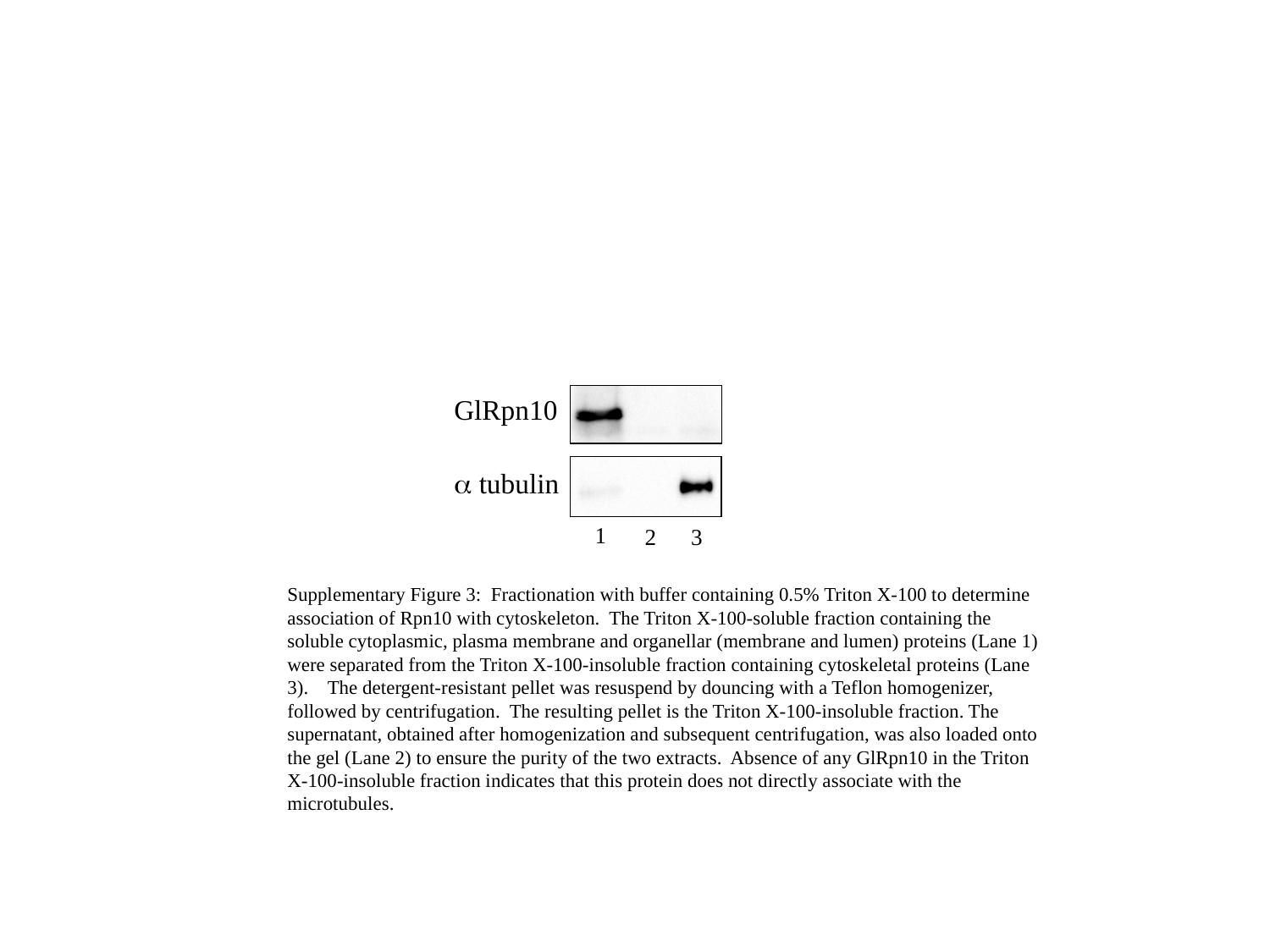

GlRpn10
a tubulin
1
2
3
Supplementary Figure 3: Fractionation with buffer containing 0.5% Triton X-100 to determine association of Rpn10 with cytoskeleton. The Triton X-100-soluble fraction containing the soluble cytoplasmic, plasma membrane and organellar (membrane and lumen) proteins (Lane 1) were separated from the Triton X-100-insoluble fraction containing cytoskeletal proteins (Lane 3). The detergent-resistant pellet was resuspend by douncing with a Teflon homogenizer, followed by centrifugation. The resulting pellet is the Triton X-100-insoluble fraction. The supernatant, obtained after homogenization and subsequent centrifugation, was also loaded onto the gel (Lane 2) to ensure the purity of the two extracts. Absence of any GlRpn10 in the Triton X-100-insoluble fraction indicates that this protein does not directly associate with the microtubules.
